# Association of thrombocytopenia with splenomegaly in malaria patients in East Kalimantan: A cross-sectional, retrospective study

**DOI:** 10.12688/f1000research.16606.3

**Published:** 2019-10-29

**Authors:** Loly R.D. Siagian, Vera M. Lumbantoruan, Nurul Hasanah, Fransiska A. Sihotang, Carta Gunawan

**Affiliations:** 1 Department of Clinical Pathology, Faculty of Medicine, Medical Faculty of Mulawarman University and Abdul Wahab Public Hospital, East Kalimantan, Indonesia; 2 Department of Dermato Venereology, Faculty of Medicine, Medical Faculty of Mulawarman University and Abdul Wahab Public Hospital, East Kalimantan, Indonesia; 3 Department of Histology, Faculty of Medicine, Medical Faculty of Mulawarman University, East Kalimantan, Indonesia; 4 Department of Physiology, Faculty of Medicine, Mulawarman University, East Kalimantan, Indonesia; 5 Department of Internal Medicine, Faculty of Medicine, Medical Faculty of Mulawarman University and Abdul Wahab Public Hospital, East Kalimantan, Indonesia

**Keywords:** thrombocytopenia, splenomegaly, malaria, East Kalimantan

## Abstract

**Background: **Malaria still presents as a major health problem in Indonesia and specifically in East Kalimantan. One common sign found in malaria patient is thrombocytopenia, the mechanism of which is still unclear. Several studies have suggested some mechanisms, one of which is splenomegaly. This study aimed to discover the association between thrombocytopenia and splenomegaly of malaria patients in East Kalimantan.

**Methods: **This study was a descriptive retrospective study with clinical and laboratory data obtained from the medical records of malaria patients in four major public hospitals from January 2015 to July 2018. The association between thrombocytopenia with splenomegaly was analysed using Chi-Square test.

**Results: **A total of 215 patients were included; 189 male (87.9%) and 26 female (12.1%). The etiologic agents found in these patients were *Plasmodium vivax *(43.2%), *Plasmodium falciparum *(42.8%), and mixed infection ( *Plasmodium falciparum *and *Plasmodium vivax*) (4.6%). The thrombocyte count was normal in 28 patients (13%) and decreased in 187 patients (87%). Among patients with thrombocytopenia, the percentage of mild, moderate and severe thrombocytopenia was 18.2%, 43.8% and 33%, respectively. Splenomegaly was found in only 11 patients (5.1%). We found no association between thrombocytopenia with splenomegaly (p=0.61).

**Conclusions: **We conclude that splenomegaly, which was a rare clinical finding of these malaria patients, was not associated with thrombocytopenia.

## Introduction

Malaria is still a serious health problem in Indonesia. The 2007 and 2013 Basic Health Surveys of the Health Ministry show that the prevalence of malaria in Indonesia increased from 2.9% to 6.0% ^1^.

Data from the East Kalimantan Provincial Health Office indicated that there were 7,045 cases of malaria in 2010. This number fluctuated in the following years, with 3,021 cases in 2011, 9,966 cases in 2012 and 2,603 cases in 2013. In terms of Annual Parasitic Incidence (API), in 2014 East Kalimantan was still above the national average with an API of 2,04 per 1000 population, leading to it being categorized as a low cumulative incidence area ^2^.

One common finding in malaria is decreased platelet count or so-called thrombocytopenia. This laboratory finding is often confused with other infectious diseases, especially dengue infection in which thrombocytopenia is a major diagnostic parameter. There are several studies that demonstrate thrombocytopenia in malaria patients ^3– 5^, and this is found in both infection with *Plasmodium falciparum* and *Plasmodium vivax*. The results from our previous study demonstrated that from 1041 malaria cases in East Kalimantan, 85% presented with thrombocytopenia of varying degrees ^4^. Therefore, thrombocytopenia has been suggested as one important diagnostic parameter in malaria. The mechanism of thrombocytopenia in malaria is still unclear. Several theories, such as mechanical trapping of thrombocytes inside the spleen and immune response that attacks thrombocytes, has been proposed ^5^. A study by Coelho *et al.* found that platelet phagocytosis may contribute to thrombocytopenia in vivax malaria ^6^.

Splenomegaly is also one common clinical finding in malaria patients. The spleen is part of the reticuloendothelial system, which becomes active in order to get rid of plasmodium-infected erythrocytes. Splenomegaly can also contribute to increased destruction of thrombocytes due to mechanical trapping. Therefore, the aim of the present study was to determine the association of thrombocytopenia with splenomegaly in malaria patients in East Kalimantan.

## Methods

### Study design and participants

This study was a cross-sectional retrospective study. This study was approved by the Ethical Committee for Health Research at Abdul Wahab Syahranie Public Hospital Samarinda, East Kalimantan (approval number 124/KEPK-AWS/V/2018). Patient consent for the use of their data records was waived by the ethical committee due to the retrospective nature of the study.

### Data collection

Data were collected between June and August 2018 from the medical records of patients with malaria during the period of January 2015 to July 2018. We collected clinical and laboratory data of both outpatients and inpatients diagnosed with malaria from four major hospitals in East Kalimantan: Abdul Wahab Sjahranie Hospital in Samarinda, Aji Putri Botung Hospital in Penajam Paser Utara, Abdul Rivai Hospital in Tanjung Redeb, and Panglima Sebaya Hospital in Tanah Grogot.

All patients with malaria, both paediatric and adult patients, were included in the study. Patients were excluded from the study if the necessary data were incomplete or patients were discharged upon own’s request during treatment.

In order to collect data, first, the hospital’s database was searched for patients diagnosed with malaria. Second, after identifying these patients, the relevant medical records were retrieved which contained age, gender, type of Plasmodium, thrombocyte count, and presence of splenomegaly. Type of Plasmodium was determined using microscopic method by obtaining thick blood smear and staining by Giemsa 3%. The thrombocyte count included in this study was from laboratory data at the time of hospital visit or admission before receiving any treatment. The thrombocyte count was determined using automated haematology analyser of different types and manufacturers available in each hospital.

### Data analysis

For descriptive data, we described patients’ characteristics that include age, sex, type of Plasmodium, and thrombocyte count on the first day admission. The association between thrombocytopenia and splenomegaly was analyzed by Chi-square test using SPSS 23.0 software. Results were considered statistically significant if p<0.05.

## Results

Our study identified a total of 215 malaria patients from January 2015 to July 2018 from four hospitals in East Kalimantan. There were 87.9% (189/215) male and 12.1% (26/215) female patients ( Table 1).

**Table 1.** Demographic characteristics of malaria patients included in the study.

Subject Characteristic	Patients (%)
Total number of patients Sex: * Male* * Female* Type of Plasmodium: *Plasmodium falciparum* *Plasmodium vivax* *Mix infection* *Unspecified Plasmodium* Thrombocyte count (per µL): *Normal (150,000-400,000)* *Thrombocytopenia (< 150,000):* Mild (100,000-150,000) Moderate (50,000-100,000) Severe (< 50,000)	215 189 (87.9) 26 (12.1) 92 (42.8) 93 (43.2) 10 (4.6) 20 (9.3) 28 (13.0) 187 (87.0) 34 (18.2) 82 (43.8) 71 (33.0)

The association of thrombocytopenia with splenomegaly in malaria patients shown in Table 2. There were 11 patients with splenomegaly and 204 patients without splenomegaly. There was no association between the presence of splenomegaly and thrombocytopenia in these malaria patients (p=0.661).

**Table 2.** Association of thrombocytopenia with splenomegaly in malaria patient.

Thrombocyte count	Splenomegaly		
	Yes	No	
Normal Thrombocytopenia Mild Moderate Severe	1 2 6 2	27 32 76 69	Chi-Square test p = 0.611
Total	11 (5.1%)	204 (94.8%)	215

Data retrieved from the medical records of malaria patients in East Kalimantan, Indonesia, including age, gender, type of Plasmodium, thrombocyte count, and presence of splenomegaly Click here for additional data file. Copyright: © 2019 Siagian LRD et al. 2019 Data associated with the article are available under the terms of the Creative Commons Zero "No rights reserved" data waiver (CC0 1.0 Public domain dedication).

## Discussion

We found that the incidence of malaria was higher in males than females in this group of patients, and the parasite infecting the majority of patients was *Plasmodium vivax* (43.2%). This study found that thrombocytopenia affected the majority of malaria patients; thrombocytopenia occurred in 87% of patients, with moderate thrombocytopenia (43.8%) as the majority. This result is in concordance with another study by Arif *et al.*, in India, that found that 79% of patients had moderate thrombocytopenia ^7^, while Ansari *et al.* found 69.18% ^8^. In the present study, the degree of thrombocytopenia classified to mild, moderate and severe was 18.2%, 43.8%, 33.0%, respectively. If we compare to study of Arif *et al.* that found 33.96%, 51.15% and 14.89%, respectively, we find that the moderate thrombocytopenia was the highest percentage in both studies ^7^.

A study by Hanson *et al.* in Vietnam found that thrombocytopenia was a marker of disease severity in adults with *Plasmodium falciparum* infection, but has limited utility in prognostication, triage and management ^9^. On the other hand, Rao *et al.* in India found that severe thrombocytopenia showed positive correlation with complicated malaria and become a good predictor for poor prognosis ^10^. Thrombocyte has an important role in haemostasis. However, study by Abro *et al.* demonstrated that malaria patients with thrombocytopenia rarely presented with any bleeding manifestations even with platelet count as low as 9,000/μL ^11^.

Indeed, splenomegaly is a hallmark of malaria, but the result of this study found that only 11 patients (5.1%) showed splenomegaly. In India, Gupta *et al.* found that only 20% malaria patients had splenomegaly, but there are no information about thrombocyte count in those malaria patients with splenomegaly ^12^.

In our study, we describe the thrombocyte count in malaria patients with splenomegaly. We found that there was no association between thrombocytopenia with splenomegaly (p=0.611). Therefore, we propose that thrombocytopenia is not caused by the mechanical trapping of thrombocytes in the spleen. This result suggests another mechanism of thrombocytopenia that involves immune process, as proposed by Coelhoe *et al.* in 2013 ^6^. Until now, definitive mechanism of thrombocytopenia in malaria is still unclear. However, some factors that contribute to thrombocytopenia have been reported, such as decreased thrombopoiesis, peripheral destruction induced by *P. falciparum* and disseminated intravascular coagulation ^11^.

This study has its limitations. Different types of automatic haematology analysers were used in the hospitals and this might contribute to variations in thrombocyte count. Therefore, we suggest the use of a single haematology analyser across hospitals to improve accuracy. In addition, we did not exclude other causes of thrombocytopenia that could coexist with malaria infection, such as viral infection and autoimmune diseases, which would be required to further validate our findings. Finally, the examination of splenomegaly was conducted by different physicians, which might contribute to subjective factors in determining splenomegaly, such as physician’s expertise and thoroughness, especially in cases with minor or subclinical splenomegaly.

Overall, we conclude that splenomegaly, which was a rare clinical finding in this set of malaria patients, was not associated with thrombocytopenia.

## Data availability

The data referenced by this article are under copyright with the following copyright statement: Copyright: © 2019 Siagian LRD et al.

Data associated with the article are available under the terms of the Creative Commons Zero "No rights reserved" data waiver (CC0 1.0 Public domain dedication). 

F1000Research: Dataset 1. Data retrieved from the medical records of malaria patients in East Kalimantan, Indonesia, including age, gender, type of Plasmodium, thrombocyte count, and presence of splenomegaly, https://doi.org/10.5256/f1000research.16606.d225124 ^13^
