# Supplementary material for: Association of thrombocytopenia with splenomegaly in malaria patients in East Kalimantan: A cross-sectional, retrospective study
Source: F1000Res. 2019 Oct 29;7:1832. Originally published 2018 Nov 21. [Version 3] doi: 10.12688/f1000research.16606.3 (PMC6823898; doi:10.12688/f1000research.16606.3)
Supplement: Peer review history

# Peer review: Reviewer response for version 3

**DOI:** 10.5256/f1000research.23205.r55902

**Published:** 2019-10-30

**Authors:** Brendan J. McMorran

**Affiliations:** 1 Department of Immunology and Infectious Disease, John Curtin School of Medical Research, Australian National University, Canberra, Australia

All my concerns have been addressed, and nothing further to add.

I confirm that I have read this submission and believe that I have an appropriate level of expertise to confirm that it is of an acceptable scientific standard.

# Peer review: Reviewer response for version 2

**DOI:** 10.5256/f1000research.22114.r54854

**Published:** 2019-10-16

**Authors:** Brendan J. McMorran

**Affiliations:** 1 Department of Immunology and Infectious Disease, John Curtin School of Medical Research, Australian National University, Canberra, Australia

The report is a relatively straight-forward and short description of a prospective malaria patient cohort, with a specific focus on platelet numbers and possible relationships between thrombocytopenia and the presence of splenomegaly. This is an important question to ask in relation to thrombocytopenia in malaria, since the mechanisms involved are not well-defined – splenic trapping is certainly a valid mechanism to consider. Their platelet data support many previous studies of other patient cohorts showing thrombocytopenia is a common clinical feature in malaria. However, the relatively small number of patients exhibiting splenomegaly (11/204 or 5.1%) precludes making a definitive conclusion about the lack of association with thrombocytopenia. Therefore, I recommend this limitation be included in the abstract and final conclusions of the paper.

 Eg. Suggested wording:

 Splenomegaly, which was a rare clinical feature of this malaria patient cohort, was not associated with thrombocytopenia.

 The methods should include notes about how platelet counts were determined, and the fact that different hematological analyzers were used (this is currently mentioned in the Discussion).

I confirm that I have read this submission and believe that I have an appropriate level of expertise to confirm that it is of an acceptable scientific standard, however I have significant reservations, as outlined above.

# Peer review: Reviewer response for version 2

**DOI:** 10.5256/f1000research.22114.r52475

**Published:** 2019-08-27

**Authors:** Jontari Hutagalung

**Affiliations:** 1 Biomedical and Basic Health Technology, National Institute of Health Research and Development, Ministry of Health (Indonesia), Jakarta, Indonesia

I confirm that I have read this submission and believe that I have an appropriate level of expertise to confirm that it is of an acceptable scientific standard.

# Peer review: Reviewer response for version 1

**DOI:** 10.5256/f1000research.18149.r40937

**Published:** 2019-06-05

**Authors:** Jontari Hutagalung

**Affiliations:** 1 Biomedical and Basic Health Technology, National Institute of Health Research and Development, Ministry of Health (Indonesia), Jakarta, Indonesia

This study is important and has a great impact for clinicians in the subject, however I found many lacks in the assessment methodologies, especially:

1. How to define the confirmed cases of this study (microscopic ELISA or PCR).
2. The methodologies to count the thrombocytopenia and splenomegaly.
3. It is important to separate the cases by species of *Plasmodium* and the day of infection.

Additional comments - there are some important things missing in this manuscript that need to be explained by the authors:

- The 1st paragraph; Please use the national Annual Parasite Incidence (API) below 1.00 per 1.000 population or the prevalence < 1%.
- The methodologies need to be addressed, because this is the most important; a) how did the authors use the definition of malaria cases in this study (giemsa 3%)? and b) for the methods of counting the parasite and thrombocytes (after treatment or before treatment, or before and after treatment), did the authors make QA for the test?
- Which prevalence of *P. vivax* is correct – 42.0% (from page 3 in the discussion) or 43.2% (from Table 1)?
- In the discussion I need more explanation about the aims of this study; "Discover the association between thrombocytopenia & splenomegaly”, please write about the following: a) the main function of thrombocytes and the kinds of situation risks decreased and for which patient conditions (acute or chronic diseases) and b) the authors should be addressing why their hypothesis was not considered significant.

Please find an annotated copy of the article here for my full comments.

I confirm that I have read this submission and believe that I have an appropriate level of expertise to confirm that it is of an acceptable scientific standard, however I have significant reservations, as outlined above.
